# Developmental cognitive genetics: How psychology can inform genetics and vice versa

**DOI:** 10.1080/17470210500489372

**Published:** 2006-09-13

**Authors:** Dorothy V. M. Bishop

**Affiliations:** University of Oxford, Oxford, UK

## Abstract

Developmental neuropsychology is concerned with uncovering the underlying basis of developmental disorders such as specific language impairment (SLI), developmental dyslexia, and autistic disorder. Twin and family studies indicate that genetic influences play an important part in the aetiology of all of these disorders, yet progress in identifying genes has been slow. One way forward is to cut loose from conventional clinical criteria for diagnosing disorders and to focus instead on measures of underlying cognitive mechanisms. Psychology can inform genetics by clarifying what the key dimensions are for heritable phenotypes. However, it is not a one-way street. By using genetically informative designs, one can gain insights about causal relationships between different cognitive deficits. For instance, it has been suggested that low-level auditory deficits cause phonological problems in SLI. However, a twin study showed that, although both types of deficit occur in SLI, they have quite different origins, with environmental factors more important for auditory deficit, and genes more important for deficient phonological short-term memory. Another study found that morphosyntactic deficits in SLI are also highly heritable, but have different genetic origins from impairments of phonological short-term memory. A genetic perspective shows that a search for *the* underlying cause of developmental disorders may be misguided, because they are complex and heterogeneous and are associated with multiple risk factors that only cause serious disability when they occur in combination.

Several developmental disorders are of particular theoretical interest to psychologists because they are characterized by fairly selective impairments in specific domains of functioning. In developmental dyslexia (also known as specific reading disability)^1^ the child has severe difficulty in learning to read, despite normal intelligence and adequate opportunity to learn. In specific language impairment (SLI) a similar profile is seen, except that oral as well as written language is affected, with comprehension as well as expression being deficient in the more severe cases. Much research on SLI has focused on the distinctive problems that children have with grammar, though in many cases other aspects of language are also impaired (L. B. Leonard, 1998). Autistic disorder involves a wider range of difficulties, encompassing poor social interaction, communication deficits, and limited repertoire of behaviour and interests, which can occur with average or even superior skills in other domains such as nonverbal reasoning and visuospatial functions. The existence of such circumscribed deficits in the context of otherwise normal development is sometimes taken as evidence for innate modular structure in the brain (see Temple, 1997, for discussion). However, in recent years this interpretation has been challenged by those who have noted that apparently selective impairments could result from a process of epigenesis, with a nonspecific deficit leading to abnormal processing of input, which has disproportionate impact on development of certain cognitive functions (Karmiloff-Smith, 1998).

In this paper, I consider what we know about causes of such disorders at two levels of description. Part 1, “How psychology can inform genetics”, focuses on studies of aetiology: genetic and environmental factors that are associated with developmental disorders. In Part 2, “How genetics can inform psychology”, attention shifts to their underlying cognitive basis. I focus mainly on SLI, but also briefly allude to relevant work on dyslexia and autism.

## How psychology can inform genetics

When neuro-imaging first became widely available, people were surprised to find remarkably little evidence of focal brain damage in children with specific developmental disorders. As C. M. Leonard (1997) remarked: “It was thought that MRI was a potential diagnostic tool—that there might be structural landmarks for each developmental disability…. There is now overwhelming evidence that children with learning disabilities do not have ‘holes in the brain’. No subsequent studies have found a one-to-one correlation between behavioral symptoms and MRI or postmortem pathology in learning disabilities” (p. 161).

As is discussed below, it is now widely accepted that inherited brain anomalies, rather than acquired brain damage, are a key factor in the aetiology of developmental dyslexia, SLI, and autistic disorder. We understand very little about how genetic variations can lead to abnormal brain development, but it is conceivable that, by affecting processes such as neuronal migration, neurotransmitter activity, programmed cell death, synaptic connectivity, or myelination, they result in a brain that functions in a nonoptimal fashion.

The bulk of the evidence for genetic aetiology of developmental disorders comes from behavioural studies adopting a genetically informative design. Such studies consider how far observed similarities between individuals (i.e., similarities in their phenotypes) are correlated with their genetic similarity (similarities in their genotypes). At the simplest level, one can do family studies to see whether a disorder is more common in first-degree relatives (parents and siblings) of an affected child than in first-degree relatives of unaffected children. Such studies are not watertight, however, because relatives typically share environments as well as genes. A more satisfactory design is the twin study, which capitalizes on the fact that there are two different processes that can lead to twinning: Splitting of a fertilized ovum will lead to genetically identical or monozygotic (MZ) twins, whereas fertilization of two ova around the same time will lead to fraternal or dizygotic (DZ) twins, whose genetic similarity is equivalent to that seen in other siblings. Although media attention focuses on studies of twins reared apart, one does not need to use such rare cases to gain useful information from a twin study. Twins growing up together will resemble one another, insofar as they are subject to many of the same environmental influences, including prenatal as well as postnatal factors. However, if similarity between two members of a twin pair is greater for MZ than for DZ twins, this points to a role of genes. When considering developmental disorders, we can assess the degree of concordance between twins (i.e., the proportion of affected cases with an affected co-twin) in both MZ and DZ twins, to get estimates of the relative roles of genetic and environmental factors in the aetiology. Studies adopting this approach have found high estimates of heritability for autism (Rutter, 2005) and SLI (Bishop, 2002). A more mixed picture has been seen for reading disability, though most studies have found sizeable genetic effects (Pennington & Olson, 2005).

**Figure 1:**
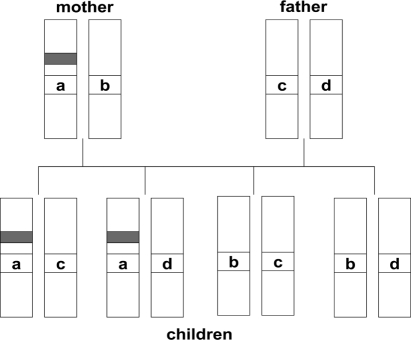
Schematic showing inheritance pattern for a small stretch of DNA. The grey region indicates an allelic variant associated with disorder. The region denoted by a, b, c, or d is a highly polymorphic noncoding region that can be used as a marker, because it is unlikely that two unrelated individuals will have identical DNA in this region. Combinations a–c, a–d, b–c, and b–d for this region are equally common in the offspring, so the a priori likelihood that any two children will have 0, 1, or 2 alleles IBD is 1 : 2 : 1. If affected sib pairs show a departure from this 1 : 2 : 1 IBD ratio, this suggests that the marker is close to an allelic variant of interest. The correlation between the marker, a, and the grey region will not be perfect, because stretches of DNA can become separated in meiosis due to crossing-over: The closer the marker is to the grey region, the more likely it is to be coinherited. In another set of families, we may see different DNA variants at this locus (w, x, y, and z instead of a, b, c, and d), but the same logic is applied: Thus the key issue is not the form that the marker takes, but rather whether it has the same parental origin in two affected sibs.

With the advent of fast-throughput genetic sequencers, a sense of optimism was generated that we would quickly move on to identify risk genes for developmental disorders. However, progress has been surprisingly slow. Identifying genes that are implicated in disorder is not a single-step procedure. Typically, one first uses a method known as linkage analysis to home in on a region that is likely to harbour relevant genes. Linkage analysis relies on the fact that the closer together two bits of DNA are, the greater the likelihood they will be inherited together. Some stretches of DNA do not contain genes and are highly variable from one individual to another. These can act as “markers”, allowing one to identify whether a portion of DNA has been inherited from the mother or the father. One starts with a set of siblings, both of whom have a disorder. Then for a pair of alleles at a given locus, one can work out whether zero, one, or two alleles in siblings are “identical by descent” (IBD)—that is, inherited from the same parent (see Figure 1). The observed IBD pattern is compared with the IBD pattern predicted by chance to identify stretches of DNA that are coinherited by affected individuals at above-expected rates. These DNA regions are likely to be close to genes important for the disorder (see Bishop, 2002, for a fuller account). Once linkage analysis has identified a marker associated with disorder, more detailed analysis of genes in that chromosomal region can be done, to look for allelic differences between affected and unaffected people. For autism, where twin studies give exceptionally strong heritability estimates, several linkages have been reported, but these have proved difficult to replicate (Barnby & Monaco, 2003). More success has been found with dyslexia (Fisher & DeFries, 2002) and SLI (Newbury, Bishop, & Monaco, 2005), but it is becoming increasingly clear that we are not going to find *the* gene for any of these disorders: They are heterogeneous, and such genes as exist are likely to act in a probabilistic fashion, in interaction with other genetic and environmental factors.

One reason why progress in the genetics of developmental disorders is slow may be because most genetic studies rely on textbook descriptions of disorders to define the phenotype. These typically specify in rather broad terms the impairments that have to be present to merit a diagnosis, together with exclusionary factors. So, for instance, SLI is diagnosed in a child whose language development lags significantly behind nonverbal development, and there is no obvious causal factor or other medical diagnosis such as hearing loss, neurological disease, or autistic disorder. This kind of diagnosis can be useful in identifying those who require special services, but it does not yield a homogenous group of children. On the one hand, it will include children with diverse difficulties in areas such as syntax, semantics, phonology, and pragmatics. On the other hand, it may exclude a child with significant language deficits because an arbitrary IQ cut-off is not met. Furthermore, although textbook definitions imply discrete disorders, the boundaries between conditions are often fuzzy—for example, SLI can be hard to distinguish from dyslexia (Bishop & Snowling, 2004) and from autistic disorder (Bishop & Norbury, 2002). There has been considerable interest in the idea that genetic discoveries may be more rapid if we use measures of underlying deficits to identify more coherent subtypes of disorder (Pennington, 1997). To date, this approach has been most widely adopted with dyslexia, but progress has been somewhat erratic. Early suggestions that deficits in phonological and orthographic processing might be linked to different genes appear to have been false positives (Fisher & DeFries, 2002); this kind of error is all too common in a field where one is conducting multiple statistical tests for linkage to many different loci. Nevertheless, there is some support for the idea that heritability may differ for different subtypes of dyslexia (Castles, Datta, Gayan, & Olson, 1999). In the field of autism, family studies suggest that we may get clearer results if we distinguish subtypes of autism with a specific language profile (e.g., Shao et al., 2002).

Identification of subtypes of disorder is one way forward, but it is not the complete solution. Rather than subdividing a disorder into ever more selective subgroups, we may sometimes need to do the opposite and consider adopting a broader conceptualization of the phenotype. The idea is not that we should simply lump together individuals with diverse clinical profiles, but rather that we should move away from a focus solely on clinical “disease” categories and develop instead more quantitative measures of underlying processes— so-called “endophenotypes” (Gottesman & Gould, 2003).

The value of this approach was demonstrated to me when I conducted a twin study of SLI (Bishop, North, & Donlan, 1995), in which we used conventional diagnostic criteria for SLI. Many twin pairs were categorized as discordant for SLI, but the “unaffected” twin often had clear evidence of language problems. In some cases, the mismatch between verbal and nonverbal skills was not great enough to merit a diagnosis of SLI, and in other cases, the child had a past history of SLI but did not show up as language-impaired on formal testing. This suggested that evidence for genetic influence on SLI would be stronger if we could use a measure of the endophenotype that captured the underlying deficits in such cases. Accordingly, we (Bishop, North, & Donlan, 1996) gave a subset of this twin sample the children's nonword repetition test (CNRep: Gathercole, Willis, Baddeley, & Emslie, 1994). The reason for choosing this measure was that Gathercole and Baddeley (1990) had found that nonword repetition was strikingly deficient in SLI, with poor performance relating to the amount of material to be remembered, rather than discrimination or production of speech sounds. They proposed that nonword repetition was a sensitive test of the phonological loop component of working memory, a key process in normal language development that was implicated in vocabulary acquisition. Like Gathercole and Baddeley (1990), Bishop et al. (1996) found major deficits in nonword repetition in children with SLI relative to control children, especially for longer nonwords. Furthermore, poor nonword repetition characterized many children who had a history of SLI, but who did not currently meet diagnostic criteria for this disorder. There were several MZ twin pairs who were concordant for poor nonword repetition, but discordant for a diagnosis of SLI. This suggested that nonword repetition might be a good behavioural marker for an underlying impairment, for which some children compensate.

**Figure 2:**
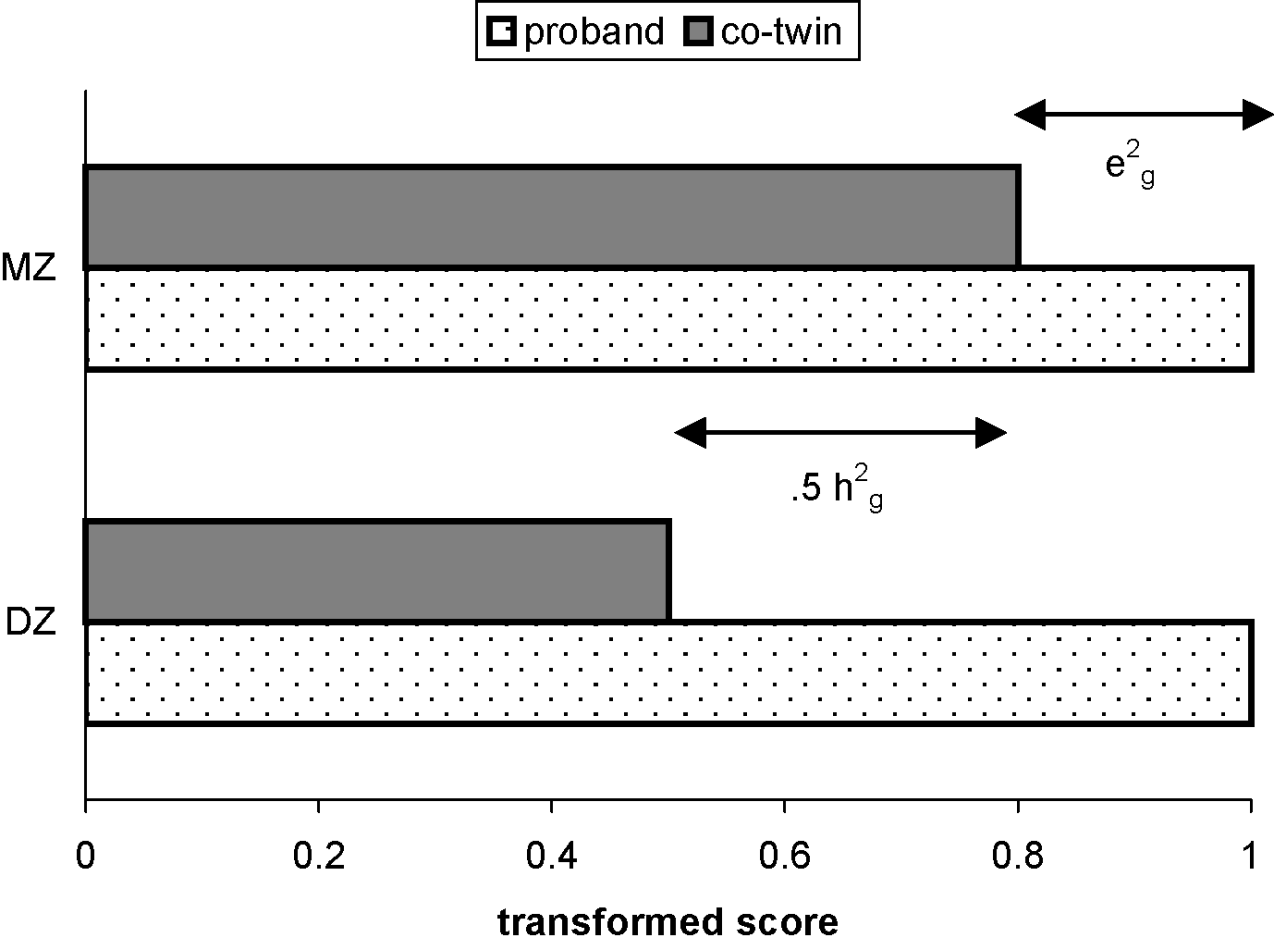
Illustration of DeFries–Fulker analysis. Data are transformed so that the population mean = 0 and the proband mean = 1. The effect of nonshared environment on impairment (*e*^2^_g_) is estimated as MZ proband mean − MZ co-twin mean. The effect of genes on impairment (*h*^2^_g_) is twice the difference between MZ and DZ co-twin means. The effect of shared environment (*c*^2^_g_) is 1−*h*^2^_g_−*e*^2^_g_.

To look at the heritability of poor nonword repetition, we used a method devised by DeFries and Fulker (1985), which was designed to estimate heritability of poor scores on a quantitative dimension. The overall logic of DeFries–Fulker analysis is parallel to more traditional twin analytic methods. Essentially, one aims to estimate the relative contribution of three potential factors that can serve to make twins more or less similar to one another. Environmental factors specific to the individual, known technically as nonshared environment or e^2^_g_, will lead to twin dissimilarity. Nonshared environment includes measurement error as well as longer term idiosyncratic influences on the child (e.g., disease that affects just one twin). If performance on nonword repetition were determined solely by e^2^_g_ then regardless of Twin A's score, we would predict that Twin B would score at the population mean. Now consider the hypothetical situation where performance on nonword repetition is determined solely by environmental factors shared by both twins—for example, the amount of language input they received from parents. Environmental factors that are common to both twins, or c^2^_g_, make twins similar to one another, so, if such factors exert a large effect, when Twin A has a low score, we predict that Twin B will also have a low score. The effect of c^2^_g_ will be the same, regardless of whether twins are MZ or DZ. Finally, consider the situation where genes, or heritable factors (h^2^_g_), are the only important influence on poor nonword repetition performance. Because MZ twins are genetically identical, if MZ Twin A has a low score, we predict that MZ Twin B will have an equally low score. However, DZ twins have, on average, 50% of segregating genes in common.^2^ For a group of DZ twins who are selected as having low scores, the prediction is that the mean score of their co-twins will regress half way to the population mean. In practice, any trait that we observe is likely to be influenced by h^2^_g_, c^2^_g_, and e^2^_g_, and our goal is to estimate the relative contribution of each of these in explaining the observed variance. Figure 2 illustrates how we can do this using DeFries–Fulker analysis. First we need to select a set of “probands”—that is, people with poor scores on our measure of interest (e.g., nonword repetition). To test the significance of the genetic term, we can use multiple regression to consider how well we can predict the scores of co-twins from the scores of probands. If the prediction is improved by including in the regression equation a term that represents the degree of genetic relationship between the twins (.5 for DZ and 1.0 for MZ), then this indicates that genes play a role in determining impairment. Bishop et al. (1996) identified probands on the basis of poor scores on CNRep, regardless of whether they met diagnostic criteria for SLI. The estimate of heritability (h^2^_g_) was close to 1.0, suggesting that genes play a major role in causing deficient phonological short-term memory (STM). These striking results from behavioural analysis have led to the inclusion of nonword repetition as a phenotypic index in molecular genetic studies. Strong linkage has been found to a site on chromosome 16 (SLI Consortium, 2002, 2004), and candidate genes in this region are now being tested for association to disorder.

The second example in this section concerns the rather discrepant findings concerning genetic influences on reading disability. Many twin studies have reported moderate to high estimates of heritability of reading disability, but there have been some notable exceptions, where environmental factors shared between twins have emerged as more important. One such case is a study by Bishop (2001b), who tested a general population sample of twin pairs and identified probands for DeFries–Fulker analysis by selecting those that scored more than 1 standard deviation below the mean on a test of nonword reading. Correlations between probands and their co-twins were high for both MZ and DZ twins, pointing to a major effect of shared environment (c^2^_g_). Estimates of genetic influence (h^2^_g_) were negligible. This was a surprising result, in the light of much higher heritability estimates for dyslexia obtained by other researchers (see Pennington & Olson, 2005, for review). However, because a previous study with an SLI group had shown close overlap between nonword repetition and literacy skills, Bishop ran an augmented version of the DeFries–Fulker analysis in which nonword repetition was included in the regression equation. This gave a significant interaction, and it was evident that for children with impaired nonword repetition, reading disability was heritable, whereas for those with normal nonword repetition skills, it was largely environmentally determined. A similar pattern of results was obtained with a younger sample of 6-year-old twins who were at the first stages of learning to read (Bishop, Adams, & Norbury, 2004a). High heritability estimates for literacy scores were found for children with poor nonword repetition, but not for those with average nonword repetition. This work has relevance for molecular genetic studies of dyslexia, because it implies that clearer genetic results will be seen if we focus attention on those poor readers with weak phonological short-term memory. Reliance on reading tests alone to identify probands will mean that we may include in our sample a substantial subset of children whose poor reading is strongly influenced by environmental risks.

The studies reviewed so far suggest that phonological short-term memory is an important component skill for language and literacy acquisition and has a strong genetic basis. Can we extend the argument further and use it as a phenotypic index in other disorders? Tager-Flusberg and Joseph (2003) noted that nonword repetition, together with certain other language skills, is frequently, though not invariably, impaired in autistic disorder, and they suggested that the same genetic factors that led to SLI in one child might lead to autism in another. A study by Bishop et al. (2004b), however, gave a different picture. In this study, probands with autism and their first-degree relatives were given a test of nonword repetition. Many of the children with autism did poorly on this test, as predicted by Tager-Flusberg and Joseph (2003). However, scores of their first-degree relatives (i.e., parents and siblings, who share 50% of segregating genes) were unimpaired, indicating that in this population, the deficit was not heritable. This contrasts with the picture in SLI and dyslexia, where relatives of affected individuals do tend to have lower scores than control populations on nonword repetition (Bishop et al., 1996; Raskind, Hsu, Berninger, Thomson, & Wijsman, 2000). It would be of interest to compare the nature of errors made on nonword repetition by children with autism with that for children with SLI, as this might point to a different underlying cause of poor performance in the two groups and so help us devise a cleaner measure of an SLI endophenotype.

In concluding this section, it is important to sound a note of caution. We hope that by improving our measures of the phenotype, we may gain new insights into the genotype, but even if we restrict attention to the much simpler case of single-gene disorders, it is clear that the relationship between genotype and phenotype is not always straightforward. The impact of a gene can be influenced by the environment to which the organism is exposed, the genetic background against which it is expressed, and random stochastic processes (U. Wolf, 1997). Environmental modulation of genetic effects is illustrated by the well-known example of phenylketonuria, where a genetic variant that usually has a detrimental effect on brain development can lead to a milder phenotype if rigorous dietary restraint is adopted (Smith, Beasley, & Ades, 1991). Another example is Huntington's disease, which, as Spires and Hannan (2005) put it, is often regarded as the “epitome of genetic determinism”: a notorious case where a single dominant allele causes a late-onset progressive neurological degeneration. This has been modelled in mice, where it has been found that the impact of the mutation can be modified by physical exercise early in life. In neurofibromatosis Type I, one can see affected relatives with the same mutation but vastly different cognitive sequelae, ranging from major mental impairment through to no detectable symptoms (Reiss & Denckla, 1996)—with phenotypic variation probably influenced by interactions between different genes (U. Wolf, 1997). And moving back to the disorders that are the focus of this paper, studies of twins make it clear that the way in which genetic risk for autism is manifest can be highly variable, even within a genetically identical MZ twin pair (Le Couteur et al., 1996). This could be due to different environmental influences on the two twins in a pair, but it could also be the result of purely stochastic influences on morphogenesis (U. Wolf, 1997).

In sum, I have given some examples from my own work showing how theoretically driven measures of underlying cognitive processes can be incorporated in a genetically informative design to help in the quest for risk genes for disorder. A major research agenda for neuropsychology is to derive better measures of such endophenotypes, rather than relying on surface manifestations of developmental disorders. It is hoped that by using quantitative measures of underlying cognitive processes rather than clinical syndromes we may find clearer relationships between phenotype and genotype. However, we must be aware that the cognitive phenotype is likely to be influenced by complex interactions between genes and environments, rather than deterministically by single genes.

## How genetics can inform psychology

Although it is still early days, most researchers interested in the causes of developmental disorders can readily see the potential of psychology for advancing progress in genetics. However, the benefits of genetic studies for informing psychology may be less immediately apparent. In the remainder of this paper, I hope to persuade readers that by using genetically informative designs we can throw new light on causal debates that have hitherto led to stalemate.

### Auditory deficit and poor phonological STM in SLI

We start by looking closely at the nonword repetition task that was the focus of the previous section. Baddeley, Gathercole, and Papagno (1998) suggested that the phonological loop component of working memory acts in humans as a “language learning device”, important for acquisition of both syntax and vocabulary, but it remains unclear how far poor phonological STM is a domain-specific linguistic deficit, or whether it is the consequence of a more general low-level perceptual impairment.

**Figure 3:**
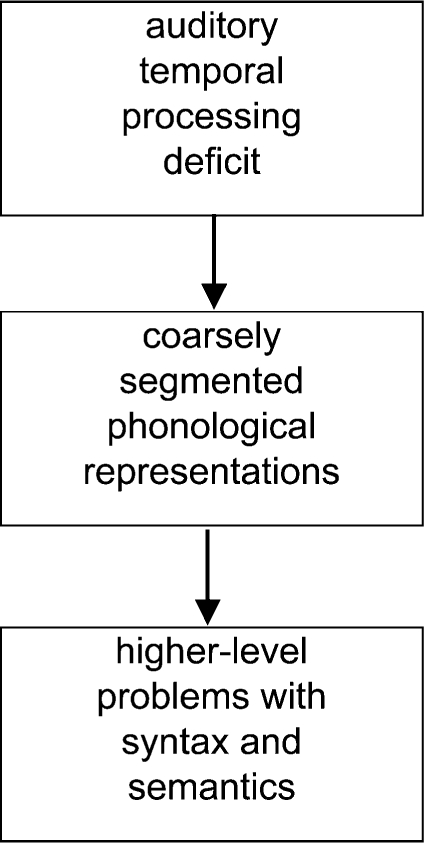
Causal route from auditory temporal processing deficit to language impairment, based on Tallal (2000).

The notion that SLI may be caused by deficient auditory perception has a long history, starting with studies by Tallal and Piercy (1973a, 1973b), who showed that children with SLI were poor at discriminating nonverbal sounds that were brief or that occurred in rapid succession. Tallal (2000, 2004) reviewed studies from a research programme that has extended over three decades, concluding that a relatively nonspecific impairment of temporal processing affects children's ability to form segmental phonological representations, leading to difficulties with mastering oral and written language. Tallal would not dispute the existence of poor phonological STM in SLI, but she would argue that this is a downstream consequence of a lower level nonlinguistic impairment (see Figure 3).

A study by Bishop, Carlyon, Deeks, and Bishop (1999b) was designed to investigate whether auditory and phonological deficits in SLI had common origins. The sample included 104 twin pairs from the general population and 37 twin pairs where one or both children had evidence of language impairment. These children were given an auditory temporal processing task (the auditory repetition test, or ART), derived from the work of Tallal, Stark, and Mellits (1985), and the CNRep, as well as a battery of expressive and receptive language tests and a test of nonverbal ability. Children with SLI were impaired on both CNRep and ART, although the effect size for the ART (expressed as proportion of variance attributed to group, η^2^ = .093) was smaller than that seen for CNRep (η^2^ = .289), and the pattern of ART errors suggested a general auditory problem, rather than a deficit specific to brief or rapid sounds. DeFries–Fulker analysis was used to analyse the heritability of deficits on both measures and yielded remarkably clear-cut results: As in the Bishop et al. (1996) study, CNRep deficits were highly heritable. However, deficits on the ART showed no genetic influence. Scores for two twins in a pair were moderately highly correlated, but this effect was comparable for MZ (*r* = .603) and DZ (*r* = .493) twins. This pattern shows that the ART is adequately reliable (if it were not, the scores of twins would not be significantly correlated) and is compatible with the test being affected by environmental influences common to both twins. In a subsequent analysis (Bishop, 2001a) it was found that musical experience in the home (as assessed by a simple questionnaire) accounted for some of this environmental effect.

**Figure 4:**
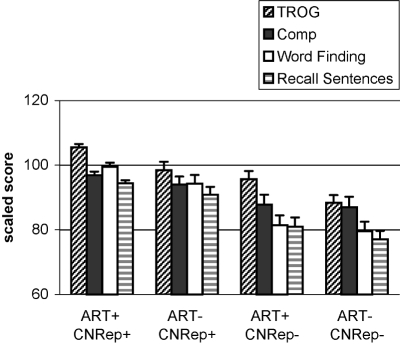
Mean scaled scores on two receptive language tests (TROG and WISC-R Comprehension) and two expressive language tests (Word Finding and CELF-R Recalling Sentences) for the sample tested by Bishop et al. (1999a), subdivided according to whether performance is more than 1 standard deviation below the mean (−), or above this level (+) on the the Auditory Repetition Test (ART) or Children's Nonword Repetition Test (CNRep).

The pattern of results was unexpected: It had been anticipated that the ART and CNRep might be different indices of the same underlying low-level auditory deficit, but the distinct aetiological influences on the two measures told a different story. One possibility that we considered was that the ART and CNRep might be indices of different subtypes of SLI, one environmentally determined and the other genetic. Although we cannot rule out this possibility, the data suggested another explanation. First, we divided up the sample into four subgroups, depending on whether they were impaired (more than 1 standard deviation below average) on the ART, the CNRep, neither, or both. The main difference between the four groups on our other language measures was in severity of language impairment, rather than pattern of strengths and weaknesses (see Figure 4). The impact of low ART and low CNRep appeared additive, with children with a double deficit obtaining the lowest language scores and being most likely to have been identified as cases of SLI. Rather than concluding that we have separate subtypes of SLI, the data suggested an explanation in terms of additive risk factors: a genetic risk, indexed by CNRep, and an environmental risk, indexed by ART.

Why, then, should we see a modest correlation (*r* = .35 after adjusting for age, IQ, and sex) between ART and CNRep, if they are not part of the same causal pathway? One possibility is that this is due to ascertainment bias: If children with A + B are more likely to have a diagnosis than children with either A or B alone, then if we pick a sample that is overrepresentative of those with a diagnosis, we see more cases than usual who have the A + B profile. Another possibility is that individuals with a genetic liability to SLI gravitate toward environments that do not favour the development of auditory processing skills in their children. They may, for instance, have less money to spend on music lessons.

**Figure 5:**
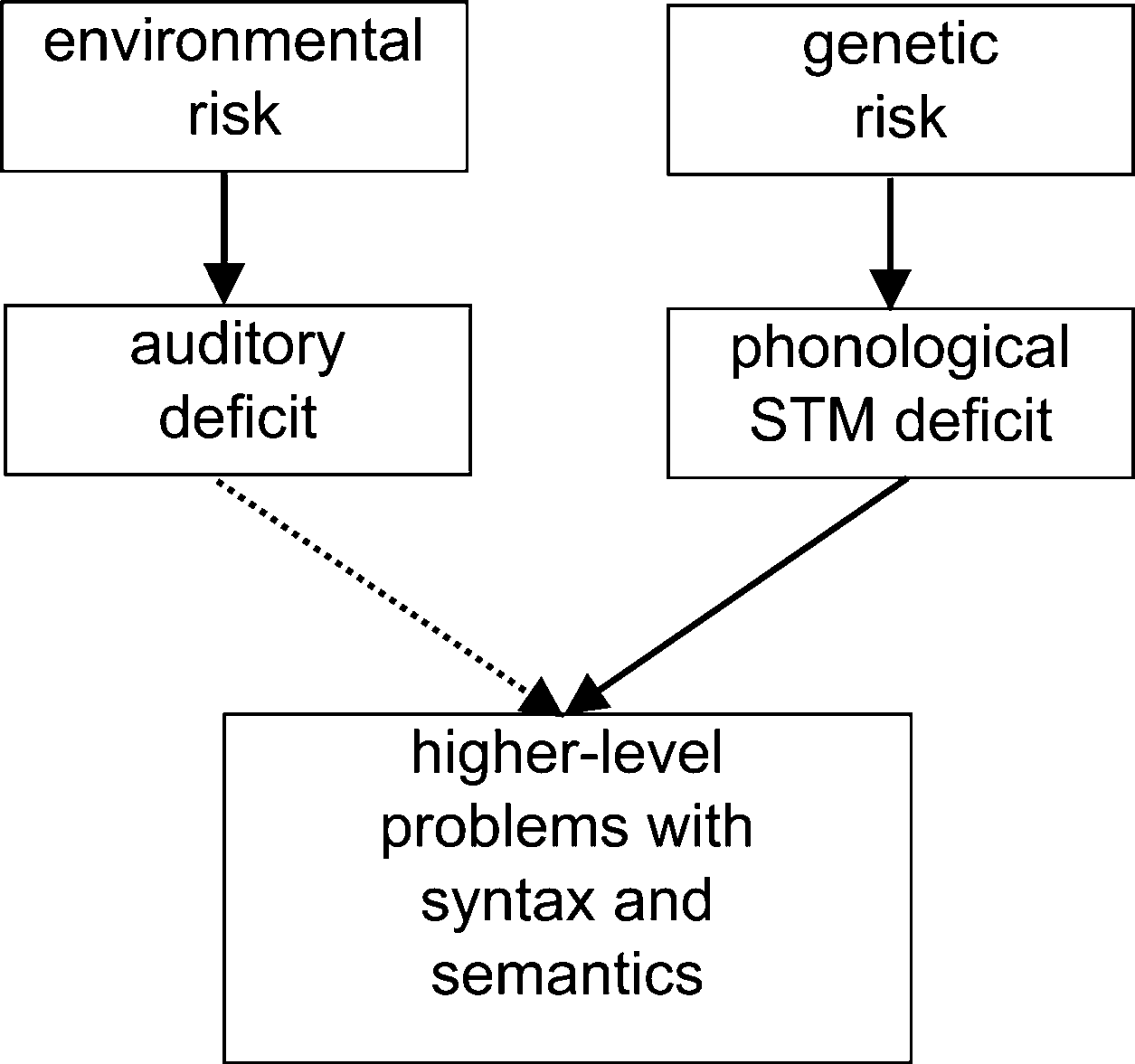
Risk factor model, in which environmental risk for language impairment is indexed by auditory deficit, and genetic risk is indexed by a deficit in phonological STM. Risk factors are probabilistic and additive, with the genetic risk having the stronger effect.

**Figure 6:**
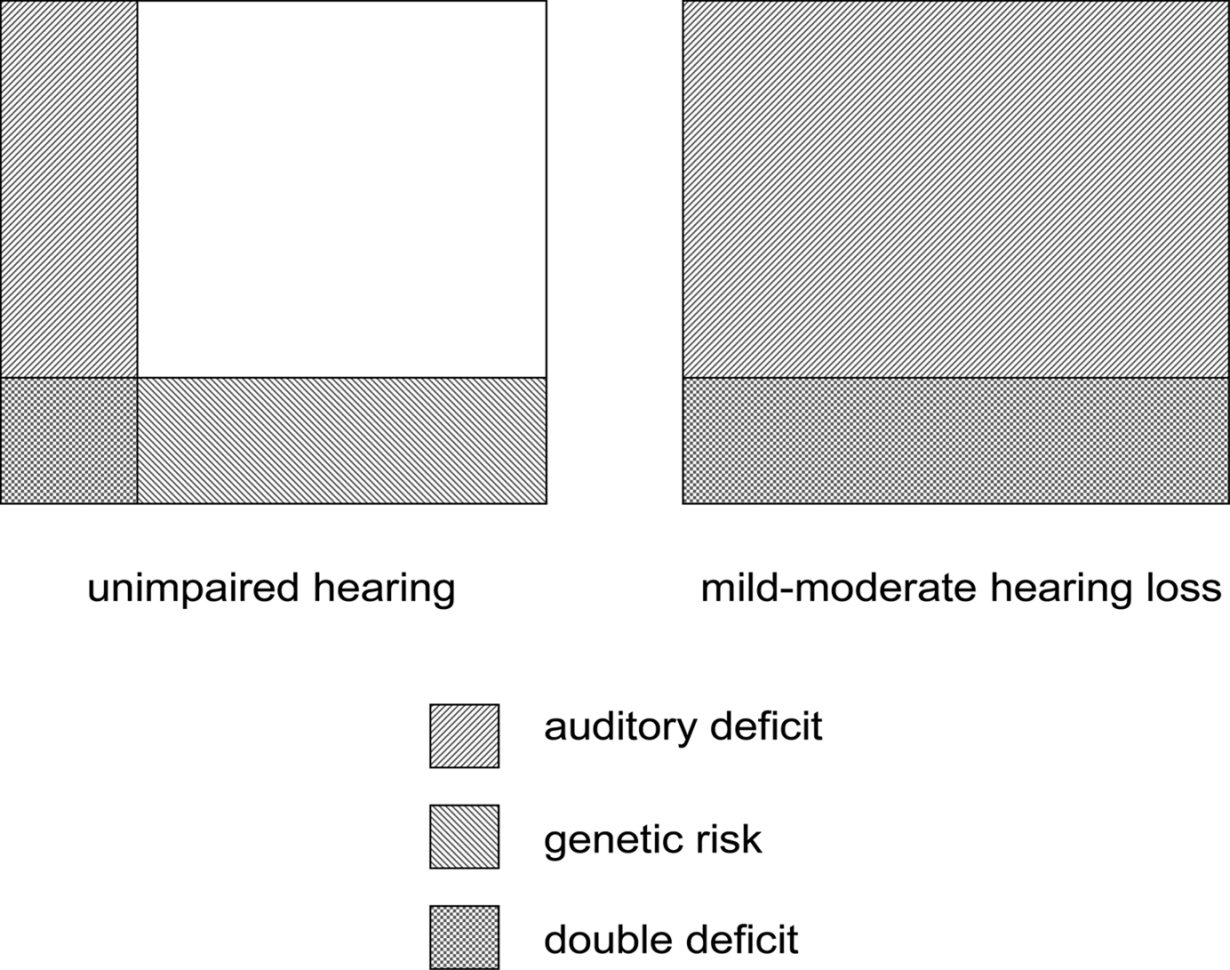
Illustration of frequency of underlying deficits in children with unimpaired hearing and those with mild–moderate hearing loss. All of the hearing-impaired group have auditory deficit; this is unlikely to manifest itself as language impairment unless a genetic risk is also present. Rates of double deficit are lower in those with unimpaired hearing.

The risk factor model (Figure 5) tells a very different story from the original auditory temporal processing account in Figure 3. Relationships between underlying deficits and SLI are seen as probabilistic: Neither genetic risk nor nonoptimal environment is necessary and sufficient to lead to SLI. Such a model is compatible with recent findings that 5-year-olds with weak phonological STM selected from the general population do not usually develop SLI (Gathercole, Tiffany, Briscoe, Thorn, & ALSPAC Team, 2005). It can also readily explain why we can find children who have deficits in ART without SLI, and children with SLI who do not have poor ART. It can also account for otherwise puzzling data obtained with children who have mild-to-moderate hearing losses in the range 20–70 dB across the speech frequencies. These children have peripheral, physical reasons for doing poorly on auditory and phonological input tasks, yet the majority of them perform in the normal range on tests of language and literacy (Briscoe, Bishop, & Norbury, 2001; Halliday & Bishop, in press; Norbury, Bishop, & Briscoe, 2001). Nevertheless, there is a small increase in risk of language impairments in this group, compared with the general population. The risk factor model makes sense of these data: Suppose around 25% of the population (both hearing impaired and normally hearing) have genetic risk for poor phonological STM, but this only has effects on language if there are also problems with auditory perception. All the hearing-impaired children with genetic risk would be expected to have language problems, but in those from the general population the genetic risk would be unlikely to be manifest as SLI unless there were also a nonoptimal environment (see Figure 6).

### Phonological STM and syntax in SLI

We have focused so far on the auditory and phonological deficits associated with SLI, omitting to mention one of the most obvious deficits seen in many children: problems with both receptive and expressive syntax (see reviews by Bishop, 1997b; L. B. Leonard, 1998). Much of the research on syntax in SLI has focused on morpho-syntax, especially use of verb inflections. For English-speaking children with SLI, one often sees a mixture of correctly inflected verbs and utterances with inflections omitted, such as:

John go there

My mummy like apples

Yesterday I walk around the castle

The problem with verb tense inflections is not confined to output, but is also seen on grammaticality judgement tasks (Rice, Wexler, & Redmond, 1999).

There are many different theoretical accounts of such deficits, but they may be broadly grouped into two camps. Those in the first camp regard SLI as a fairly specific deficit affecting syntax acquisition. In one of the more influential versions of this type of theory, Rice, Wexler, and Cleave (1995) proposed an extended optional infinitive account of SLI. According to this account, appropriate marking of infinitives is a function of maturation. Before 4 years of age, most children behave as if such marking is optional. By around 4 years of age their grammar matures, and infinitive marking is treated as obligatory. In SLI, maturation is delayed and children continue to treat infinitive marking in English as optional, and hence they frequently omit verb tense markers in contexts where they are obligatory.

Theorists in the second camp argue instead that the linguistic deficits in SLI may be the consequence of poor auditory perception (e.g., Bates, 2004; Bishop, 1997b), limitations of processing capacity (e.g., Bishop, 1994), phonological deficits (Joanisse, 2004), or some combination of these (L. B. Leonard, 1998). In each case, the argument is that some aspects of syntax will be especially vulnerable, because they are marked by phonetically weak segments, and/or place particularly heavy demands on processing capacity when comprehending or producing language. According to this type of theory, syntactic deficits and phonological deficits in SLI have similar origins.

It has been difficult to decide between theories on the basis of correlational evidence alone, not least because one is never clear whether imperfect correlations between syntactic and other deficits are due to poor reliability of measurement, extraneous task demands, or measurements being taken at an inappropriate age. A simulation study by Joanisse and Seidenberg (2003) demonstrated that perceptual limitations could plausibly lead to syntactic comprehension deficits, and Hayiou-Thomas, Bishop, and Plunkett (2004) found that typically-developing children produced similar responses to those seen in SLI on a grammaticality judgement task when difficulty was increased by either compressing the speech or increasing memory load. Such studies demonstrate how syntactic deficits could plausibly arise because of lower level deficits, but this does not, of course, prove that this is the mechanism at work in SLI.

The opportunity to address this issue in a genetically informative design arose in the course of a study of 173 twin pairs at the age of 6 years (Bishop, Adams, & Norbury, in press). The twin sample was a subset of children from the Twins Early Development Study (Trouton, Spinath, & Plomin, 2002); two thirds were in a “language risk” group—that is, they had been selected because one or both had evidence of language difficulties at 4 years of age; the remainder (the “low risk” group) were selected at random from those without evidence of language difficulty. Children were given a battery of measures of language and nonverbal ability, as well as the CNRep, and two subtests that were designed to elicit verb inflectional endings: the third-person singular and past-tense probes from the Rice–Wexler Test of Early Grammatical Impairment (Rice & Wexler, 2001).

The CNRep was less good at discriminating children with language difficulties than it had been in older children (effect size, η^2^ = .10) but the data suggested that this might be because of articulatory difficulties. We derived a measure that was intended to act as a purer index of phonological STM by using a regression equation to compute the mismatch between the score obtained with three- to five-syllable nonwords and that predicted from the two-syllable score. This phonological STM score did a better job than the raw nonword repetition total at discriminating children in the “language risk” group from the “low risk” group (η^2^ = .15), and it was significantly heritable (*h*^2^_g_ = .61) on DeFries–Fulker analysis.

The Rice–Wexler subtests were combined to give a single index of verb inflections. This was highly skewed, consistent with the extended optional infinitive theory, which maintains that most children have mastered this aspect of grammar by 4 years of age. The skewing does not affect results from DeFries–Fulker analysis and may indeed be a useful indicator of a major gene effect (Bishop, 2005). Heritability of poor performance on verb inflections was high (*h*^2^_g_ = .74).

The next question was whether the two tests were different markers of the same underlying problem, as suggested by those theorists who attribute syntactic deficits to problems with phonological processing. The association between the two deficits was significant but not high: 14% of the sample were impaired on phonological STM only, 11% on verb inflections only, and 9% on both: φ = .257, *p* < .01. The fact that both measures were significantly heritable suggests that this lack of agreement is not readily explained by poor test reliability, as an unreliable test would simply show up on DeFries–Fulker analysis as having a large *e*^2^_g_ term. To investigate the extent of aetiological overlap between the measures, a bivariate extension of DeFries–Fulker analysis was used. This involves predicting the score of twin B on test Y from the score of twin A on text X, after scaling both measures appropriately (Purcell et al., 2001). If the prediction is improved by including the genetic relationship between the twins in the equation, then this points to some genetic overlap in the aetiology of the two deficits. In this instance, the parameter *h*^2^_g.xy_ was close to zero. This means that for probands who were low on X, there was no evidence for genetic factors that also caused low Y.

These results were surprising and challenge those who see syntactic deficits and phonological problems as having common origins. Supplementary analyses of the data ruled out alternative explanations of poor syntax in terms of weak vocabulary, poor articulation, or low IQ, suggesting that this is a rather specialized area of impairment. Interestingly, one other language test was highly heritable and did show genetic overlap with the verb inflections measure, and this was a test of sentence comprehension, CELF-R Sentence Structure (Semel, Wiig, & Secord, 1987), in which the task is to select from syntactic and semantic foils a picture that matches a spoken sentence. This suggests that the genes that affect verb inflectional skill may have broader effects on computation of syntactic relationships between sentence elements, as proposed by Van der Lely (2005).

This study threw up the same puzzle as did the earlier work with the ART—namely, how to explain the fact that different areas of language deficit (in this case phonological STM and verb inflections) were significantly, albeit weakly, correlated, when the twin analysis suggested that they had different origins. As with the previous work, one interpretation is in terms of additive risk factors; children with a double deficit may be more likely to have clinically significant language problems and hence find their way into a study such as this. Given that both of the measures were heritable, another possibility is that there may be assortative mating for language disorder, so that parents with either kind of impairment tend to have children together. To test this idea we would need to assess parents directly.

## Causality in developmental disorders: Insights from behaviour genetics

Overall, these two twin studies (Bishop et al., 1999a, in press) demonstrate the power of genetically informative designs to clarify relationships between different areas of impairment in developmental disorders. When we do so, we find a surprising degree of aetiological separation between different impairments that are associated with SLI. Deficits of low-level auditory perception (as measured by the ART) appear environmentally determined. Both phonological STM and syntactic deficits are heritable, with little evidence of any environmental influence, but different genes appear to be implicated in the two areas of difficulty. This kind of evidence appears to sit nicely with the “double dissociation” logic of developmental cognitive neuropsychology, in showing that different cognitive components are separable, not just in terms of phenotypic manifestations, but also in terms of underlying aetiology.

There is, however, another aspect of the data that is typically ignored by those working in the cognitive neuropsychology framework, and that is the finding that although specific deficits in developmental disorders are dissociable, they are nevertheless often associated. Associations are often seen as theoretically uninformative because, as every student learns in Psychology 101, correlation does not imply causation. Indeed, in the quest for necessary and sufficient causes of disorder, one case of dissociation between deficits is regarded as providing far more powerful evidence than numerous cases of association: Correlation does not imply causation, but lack of correlation is seen as *disproving* causation. Quite simply, if one can find a child who has grammatical deficits in the absence of auditory impairment, then this is evidence that the grammatical deficits are not caused by auditory impairment (cf. Temple, 1997; Van der Lely, Rosen, & Adlard, 2004). As Bishop (1997a) and Karmiloff-Smith (1998) have noted, the argument is not entirely watertight, because one needs to take a developmental perspective and be aware that perceptual deficits at an early stage in development might resolve, but nevertheless affect subsequent development of the cognitive system. But even leaving this argument to one side, there remains a question that is unresolved by those who focus solely on dissociation in developmental disorders, and that is the need to explain the occurrence of reliable associations between different types of impairment. Quite simply, it is all very well to note that grammatical deficits can occur without auditory impairments, but we still need to explain why auditory impairments are relatively common in children with grammatical deficits. Ramus (2004) has made a similar point in the context of sensorimotor deficits associated with developmental dyslexia.

We need to be alert to the possibility that such associations may be artefactual (due either to sampling bias or to specific task demands), but a more interesting possibility is that probabilistic associations between deficits are telling us something about causality. Most readers will agree that smoking causes lung cancer. We are willing, when arguing about the aetiology of this physical disease, to think of smoking as a risk factor that increases the risk of lung cancer, while accepting that the association between cause and disease is probabilistic, rather than deterministic. It would be a serious mistake to dismiss any role for smoking in causing lung cancer, because Uncle Fred smoked all his life and lived to a ripe old age, or Auntie Susan never smoked but died from lung cancer.

A recurring finding in developmental disorders is that underlying deficits can be dissociated, but when they co-occur the disorder is more severe and more likely to be detected. In the case of SLI we found that weak phonological STM and auditory impairment were aetiologically as well as phenotypically dissociable, but when both deficits occurred together, the child was particularly likely to receive a clinical diagnosis. In a similar vein, syntactic and phonological impairments were genetically distinct and were often dissociated, but it was the children with both problems who had the most severe language difficulties (Bishop et al., in press). Such results suggest that children's language development is generally resilient in the face of a specific cognitive deficit, but that where two or more such deficits occur together, their effect is additive, and the chances increase that language learning will break down.

Similar arguments have been put forward in the domains of developmental dyslexia and autism. For dyslexia, M. Wolf et al. (2002) proposed the notion of a “double deficit”, with independent problems in phonological awareness and naming speed affecting literacy acquisition. They showed that those with the most severe and intractable reading difficulties were most likely to have a double deficit. Pennington (in press) has tackled similar issues with the aim of explaining comorbidities between dyslexia and attention deficit hyperactivity disorder on the one hand, and speech-sound disorder on the other hand. He concluded that the traditional cognitive neuropsychology approach of using double dissociations to identify a single underlying deficit in dyslexia could not explain the pattern of associations and dissociations that is observed between disorders, and he proposed a multiple deficit model that regards dyslexia as the result of multiple risk and protective factors, both environmental and genetic.

Autistic disorder is diagnosed on the basis of there being a triad of impairments affecting socialization, communication, and restricted interests/behaviour, but there is growing awareness that these areas of impairment can be dissociated from one another in children who fall short of meeting diagnostic criteria (e.g., Bishop & Norbury, 2002), and those working on the genetics of autism are beginning to consider whether different dimensions of deficit may be influenced by different genes (Folstein, Dowd, Mankoski, & Tadevosyan, 2003; Ronald, Happé, & Plomin, 2005). In autism, there is evidence that different risk factors may have a synergistic rather than merely additive impact. Among relatives of affected people, one can find individuals with one or two of the cognitive features that characterize autism who function normally in everyday life. However, when several such features co-occur in the same individual, there is a catastrophic impact, leading to a diagnosis of autism (Bailey & Parr, 2003). Such evidence suggests that children may be able to employ different pathways to success in language, literacy, and social interaction and only come adrift if multiple deficits shut off alternative routes to success.

In sum, cognitive studies of developmental disorders have often embraced parsimony and have looked for a single necessary and sufficient “cause” of disorders such as dyslexia, SLI, and autism. I suggest that this approach has led to theoretical stagnation, with much pointless debate about whether, for instance, auditory perceptual deficits are *the* cause of dyslexia or SLI. As Bishop et al. (1999b) argued, the evidence indicates that auditory deficits are neither necessary nor sufficient for causing SLI, but this does not mean that they play no role. A “single cause” approach is too simple to account for the clinical reality. Identifying risk factors, and determining how they operate together, may be a more fruitful approach.

## Footnotes

1 The terminology for such disorders is a contentious topic, with some regarding “developmental dyslexia” as misleading in implying a clinical syndrome. Specific reading disability (SRD) is preferable as a neutral description that carries less theoretical baggage, but it has the disadvantage of being more wordy and less widely understood than “dyslexia”.

2 Nongeneticists may find this puzzling, given the popular statistic that humans share over 98% of their genes with chimpanzees! In the behaviour genetics context, we are interested only in polymorphic or segregating genes—that is, coding regions that can take different allelic forms in different people, as these are the only regions of DNA that can cause individual differences. These are only a tiny proportion of the human genome.
